# *QuickStats:* Percentage* of Adults Aged ≥18 Years Who Received Care at Home From a Friend or Family Member in the Past 12 Months,^†^ by Sex and Age Group — National Health Interview Survey,^§^ United States, July–December 2020

**DOI:** 10.15585/mmwr.mm7102a5

**Published:** 2022-01-14

**Figure:**
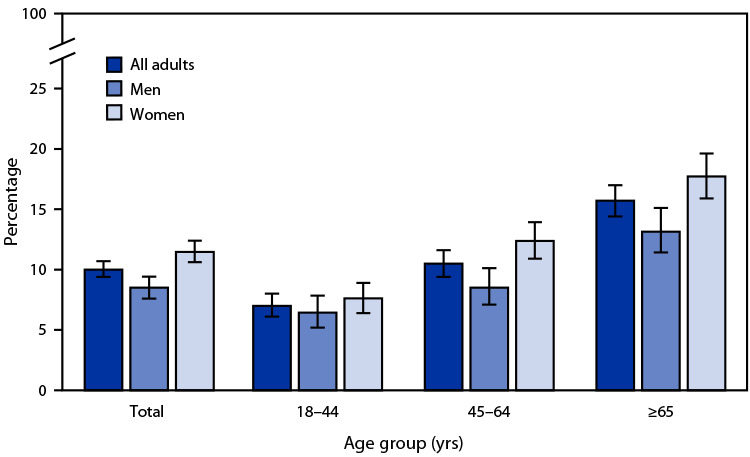
During July–December 2020, 10.0% of adults aged ≥18 years received care at home from a friend or family member in the past 12 months. Among both men and women, the percentage of adults who received care in the past 12 months increased with age. Women were more likely than men to receive care among those aged ≥18 years (11.5% and 8.5%, respectively), 45–64 years (12.4% and 8.5%, respectively), and ≥65 years (17.7% and 13.2%, respectively).

**Source:** National Center for Health Statistics, National Health Interview Survey, 2020. https://www.cdc.gov/nchs/nhis/index.htm

